# Virulence gene profiles of avian pathogenic *Escherichia coli* isolated from chickens with colibacillosis in Bulawayo, Zimbabwe

**DOI:** 10.4102/ojvr.v82i1.850

**Published:** 2015-04-07

**Authors:** Joshua Mbanga, Yvonne O. Nyararai

**Affiliations:** 1 Department of Applied Biology and Biochemistry, National University of Science and Technology, Zimbabwe

## Abstract

Colibacillosis, a disease caused by avian pathogenic *Escherichia coli* (APEC), is one of the main causes of economic losses in the poultry industry worldwide. This study was carried out in order to determine the APEC-associated virulence genes contained by *E. coli* isolates causing colibacillosis in chickens. A total of 45 *E. coli* isolates were obtained from the diagnostics and research branch of the Central Veterinary Laboratories, Bulawayo, Zimbabwe. These isolates were obtained from chickens with confirmed cases of colibacillosis after postmortem examination. The presence of the *iutA*, *hlyF*, *ompT*, *frz*, *sitD*, *fimH*, *kpsM, sitA*, *sopB, uvrY,* *pstB* and *vat* genes were investigated by multiplex polymerase chain reaction (PCR) assay. Of the 45 isolates, 93% were positive for the presence of at least one virulence gene. The three most prevalent virulence genes were *iutA* (80%), *fimH* (33.3%) and *hlyF* (24.4%). The *kpsM,* *pstB* and *ompT* genes had the lowest prevalence, having been detected in only 2.2% of the isolates. All 12 virulence genes studied were detected in the 45 APEC isolates. Virulence gene profiles were constructed for each APEC isolate from the multiplex data. The APEC isolates were profiled as 62.2% fitting profile A, 31.1% profile B and 6.7% profile C. None of the isolates had more than seven virulence genes. Virulence profiles of Zimbabwean APEC isolates are different from those previously reported. Zimbabwean APEC isolates appear to be less pathogenic and may rely on environmental factors and stress in hosts to establish infection.

## Introduction

In chickens, colibacillosis refers to any local or systemic infection caused entirely or partially by *Escherichia coli* (Omer *et al*. 2010). Colibacillosis is economically important worldwide as it results in high morbidity and mortality of affected birds (Dho-Moulin & Fairbrother 1999; Dziva & Stephens 2008). The infection is generally initiated or enhanced by predisposing agents, such as mycoplasmal or viral infections and environmental factors (Dho-Moulin & Fairbrother 1999). However, management approaches based only on protecting poultry against predisposing conditions have proved largely ineffective for controlling outbreaks of avian colibacillosis (Barnes, Nolan & Vaillancourt 2008).

Antimicrobial drugs remain important in reducing both incidence and mortality associated with this disease (Zakeri & Kashefi 2012) but there is increasing evidence that avian pathogenic *E. coli* (APEC) is becoming more resistant to antimicrobial agents (Saidi, Marifakureva & Mbanga 2013; Sharada, Ruban & Thiyageeswaran 2009; Skyberg *et al*. 2006). A vaccine based approach for the control of outbreaks of avian colibacillosis is therefore highly desirable (Olsen, Christensen & Bisgaard 2012). Currently available vaccines are not totally effective (Schouler *et al*. 2012). This is mainly because of the diverse characteristics of APEC strains, which prevent the identification of common properties that could be used as a basis for vaccination (Schouler *et al*. 2012).

Several virulence factors have been associated with the virulence of APEC, including those encoding for adhesins, toxins, iron acquisition systems, autotransporters, sugar metabolism, serum resistance proteins, and capsule as well as lipopolysaccharide complexes (Ewers, Janssen & Wieler 2003; Li *et al*. 2005; Schouler *et al*. 2012). However, numerous studies have demonstrated that these virulence factors are rarely all present in the same isolate and that they can occur either individually or polygenically with varying frequencies in clinical isolates (Delicato *et al*. 2003; Vandekerchove *et al*. 2005). This all indicates that APEC strains constitute a heterogeneous group. The accurate identification of virulent strains of *E. coli* and the virulence genes they possess is essential if genes that can serve as vaccine targets are to be identified. Virulence gene studies are therefore important as they not only aid in the characterisation of pathogenic strains of *E. coli* but may eventually lead to the development of effective vaccines.

Work on virulence genes in the western world and most of Asia is on the rise, but in Africa very little information seems to be available (Randall *et al*. 2012; Van der Westhuizen & Bragg 2012). In this study, 12 different APEC virulence genes were used to characterise Zimbabwean APEC isolates. The 12 genes used in this study included the *iutA*, *hlyF*, *ompT*, *frz*, *sitD*, *fimH*, *kpsM, sitA*, *sopB, uvrY,* *pstB* and *vat* genes (full names provided in Table 2). These have all been previously reported in literature, and some have been shown to occur in high frequencies in APEC isolates from other countries (Johnson *et al*. 2008; Kafshdouzan *et al*. 2013; Schouler *et al.* 2012). A number of studies have reported the successful use of multiplex polymerase chain reaction (PCR) in the detection of virulence genes in *E. coli* isolated from different organisms like dogs (Pass, Odedra & Batt 2000), pigs (Schierack *et al*. 2006) and poultry (Ewers *et al*. 2005). This study used multiplex PCR developed by Van der Westhuizen and Bragg (2012) to detect virulence genes in APEC isolates. Virulence gene profiles were then constructed for each APEC isolate from the multiplex data. This study was carried out in order to provide information on the virulence factors of *E. coli* isolated from chickens with confirmed cases of colibacillosis in Zimbabwe.

## Materials and methods

### Sample collection

All of the 45 *E. coli* isolates used in this study were obtained from the diagnostics and research branch of the Central Veterinary Laboratories, Bulawayo. These isolates were obtained from chickens with confirmed cases of colibacillosis after postmortem examination. Confirmatory tests were carried out on the isolates and the identification of *E. coli* was performed according to methods described by Barrow and Feltham (1993). Biochemical tests included the Gram stain and the catalase, oxidase, indole and citrate tests.

### DNA extraction

Bacterial strains were subcultured overnight at 37 °C in Luria-Bertani (LB) broth (Oxoid, Basingstoke, Hampshire, UK) and genomic deoxyribonucleic acid (DNA) was extracted using a standard phenol-chloroform method (Sambrook & Russell 2001). To check for purity, DNA was run along a 1% ethidium bromide–stained agarose gel (Sigma-Aldrich, St Louis, USA) with a 1 kb DNA ladder (Thermo Scientific, Waltman, Massachusetts, USA) in tris-borate-EDTA (TBE) buffer for 1 hr at 100 V and then viewed using the Uvipro Silver Gel Documentation System (Uvitec, Cambridge, UK). The concentration of DNA was estimated by comparing the band light intensity to the band intensity on the 1 kb ladder on the Uvipro Silver Gel Documentation System. DNA concentration of samples ranged from 75 ng/0.5 µg to 100 ng/0.5 µg.

### Virulence genotyping

The presence of genes encoding virulence factors was detected using multiplex PCR amplification. Four multiplex PCR assays were used to detect 12 virulence genes (Table 1). The multiplex design was according to that reported by Van der Westhuizen and Bragg (2012), with slight changes in the primer and final magnesium chloride concentrations. The effected changes were using primer concentrations of 0.5 µM for the *frz*, *sitD*, *fimH*, *ompT*, *iutA*, *pstB* and *sopB* genes and adjusting the final magnesium chloride (MgCl_2_) concentration to 3 mM for all multiplex reactions. The primers used in this study are listed in Table A1 in Appendix 1. All primers used were obtained from Inqaba Biotech, Pretoria, South Africa. Three microlitres of each of the DNA samples were mixed with all necessary components for amplification in a 0.2 mL PCR tube (Perkin-Elmer, Waltman, Massachusetts, USA) in a 25 µL reaction. The reaction mixture included 2.5 µL of ×10 PCR Dream Taq buffer (Thermo Scientific, Waltman, Massachusetts, USA), 2 µL of deoxynucleotide triphosphates (dNTPs) 10 mM; 0.25 µL of Dream Taq polymerase (Thermo Scientific, Waltman, Massachusetts, USA), 5U/µL nuclease-free water to maintain a total volume of 25 µL. The appropriate primers ranging from 0.5 µM to 2 µM were added and the MgCl_2_ concentration was adjusted to a final concentration of 3 mM, as shown in Table 1. Negative controls comprised a water control. An Applied Biosystems GeneAmp^®^ PCR System 9700 was used for the PCR thermal cycling conditions with an initial denaturation step at 94 °C for 5 min, 35 cycles (denaturation at 94 °C for 30 sec, annealing at 63 °C for 45 sec, extension at 72 °C for 1 min and 45 sec) and a final elongation step at 72 °C for 10 min. The amplified products were then run along a 1% ethidium bromide–stained agarose gel with a 100 bp DNA ladder (Thermo Scientific, Waltman, Massachusetts, USA) in TBE buffer for 1 hr at 100 V and then viewed using the Uvipro Silver Gel Documentation System (Uvitec, Cambridge, UK).

**TABLE 1:** Final primer concentrations used in the different multiplex polymerase chain reactions.

Multiplex	Primer set	Concentration (μM)	Primer set	Concentration (μM)	Primer set	Concentration (μM)	Additional MgCl2 (mM)	Final MgCl2 (mM)
1	*frz*	0.5	*sitD*	0.5	*fimH*	0.5	1	3
2	*sitA*	2.0	*kpsM*	1.0	*Vat*	0.5	1	3
3	*ompT*	0.5	*iutA*	0.5	*pstB*	0.5	1	3
4	*sopB*	0.5	*uvrY*	1.0	*hlyF*	0.5	1	3

MgCl_2_, Magnesium chloride.

The multiplex PCRs described were used to screen for the presence of 12 virulence genes in the APEC isolates in duplicate. Prevalence of each virulence gene was calculated (Table 2) and virulence gene profiles were then assigned to each APEC isolate (Table 3).

**TABLE 2:** Frequency of the 12 virulence genes in 45 avian pathogenic *Escherichia coli* isolates.

Name of gene	Primer name	Frequency (%)
Aerobactinsiderophore receptor	*iutA*	80
Type 1 fimbrial adhesin	*fimH*	33.3
Vacuolating autotransporter toxin	*vat*	17.8
*SitABCD*system	*sitA*	11.1
*SitABCD*system	*sitD*	13.3
Putative avian haemolysin	*hlyF*	24.4
*PstSCAB*system	*pstB*	2.2
*frz*operon	*frz*	8.9
APEC virulence regulator	*uvrY*	4.4
Capsule-protein transport of polysaccharides	*kpsM*	2.2
Episomal outer membrane protease	*ompT*	2.2
Plasmid partitioning protein	*sopB*	20

**TABLE 3:** Presence or absence of expected amplicons and virulence profiles of avian pathogenic *Escherichia coli* isolates.

Isolate	Multiplex 1			Multiplex 2			Multiplex 3			Multiplex 4			Total/12	Virulence profile
*frz*	*sitD*	*fimH*	*sitA*	*kpsM*	*vat*	*ompT*	*iutA*	*pstB*	*sopB*	*uvrY*	*hlyF*
CVL1	−	−	−	−	−	−	−	−	−	−	−	−	0	A†
CVL2	−	−	−	−	−	−	−	−	−	+	−	+	2	A
CVL3	−	−	+	−	−	−	−	+	−	+	−	+	4	B‡
CVL4	−	−	−	−	−	−	−	+	−	+	−	+	3	B
CVL5	−	−	+	−	−	−	−	−	−	+	−	+	3	B
CVL6	−	−	+	−	−	−	−	+	−	+	−	+	4	B
CVL7	−	−	−	−	−	−	−	+	−	+	−	+	3	B
CVL8	−	−	−	−	−	−	−	+	−	−	−	−	1	A
CVL9	−	−	+	−	−	−	−	−	−	+	+	+	4	B
CVL10	−	−	−	−	−	−	−	−	−	+	−	+	2	A
CVL11	−	−	−	−	−	−	−	+	−	−	−	−	1	A
CVL12	−	−	−	−	−	+	−	−	−	−	−	−	1	A
CVL13	−	−	−	−	−	−	−	+	−	−	−	−	1	A
CVL14	+	+	+	+	−	+	−	+	−	−	−	−	6	C§
CVL15	+	+	+	+	−	+	−	+	−	−	−	−	6	C
CVL16	+	+	+	+	+	+	−	+	−	−	−	−	7	C
CVL17	−	+	+	+	−	+	−	+	−	−	−	−	5	B
CVL18	−	−	−	−	−	−	−	−	−	−	−	−	0	A
CVL19	−	−	+	−	−	−	−	+	−	−	−	−	2	A
CVL20	−	−	−	−	−	−	−	+	+	−	−	−	2	A
CVL21	−	−	+	−	−	−	−	+	−	−	−	−	2	A
CVL22	−	−	+	−	−	−	−	+	−	−	−	−	2	A
CVL23	−	−	−	−	−	−	−	+	−	−	−	−	1	A
CVL24	−	−	−	−	−	−	−	+	−	−	−	−	1	A
CVL25	−	−	−	+	−	+	−	+	−	−	−	−	3	B
CVL26	−	−	−	−	−	−	−	−	−	−	−	−	0	A
CVL27	−	−	−	−	−	−	−	+	−	−	−	−	1	A
CVL28	−	−	−	−	−	+	−	+	−	−	−	−	2	A
CVL29	−	−	−	−	−	−	−	+	−	+	−	+	3	B
CVL30	−	−	−	−	−	+	−	−	−	−	−	−	1	A
CVL31	−	−	−	−	−	−	−	+	−	−	−	−	1	A
CVL32	−	−	−	−	−	−	−	+	−	−	−	−	1	A
CVL33	−	−	−	−	−	−	−	+	−	−	−	−	1	A
CVL34	−	+	+	−	−	−	−	+	−	−	−	−	3	B
CVL35	−	−	−	−	−	−	−	+	−	−	−	−	1	A
CVL36	+	−	+	−	−	−	−	+	−	−	−	−	3	B
CVL37	−	−	−	−	−	−	+	+	−	−	−	+	3	B
CVL38	−	−	−	−	−	−	−	+	−	−	−	−	1	A
CVL39	−	−	−	−	−	−	−	+	−	−	−	+	2	A
CVL40	−	−	−	−	−	−	−	+	−	−	−	−	1	A
CVL41	−	+	+	−	−	−	−	+	−	−	−	−	3	B
CVL42	−	−	−	−	−	−	−	+	−	−	−	−	1	A
CVL43	−	−	+	−	−	−	−	+	−	−	+	−	3	B
CVL44	−	−	−	−	−	−	−	+	−	−	−	−	1	A
CVL45	−	−	−	−	−	−	−	+	−	−	−	−	1	A

CVL, Central Veterinary Laboratories isolate obtained from confirmed case of colibacillosis.

+, Presence of the expected amplicon during multiplex polymerase chain reaction; -, absence of the expected amplicon during multiplex polymerase chain reaction; †, virulence profile A indicates presence of 0 to 2 virulence genes; ‡, virulence profile B indicates presence of 3 to 5 virulence genes; §, virulence profile C indicates presence of 6 or more virulence genes.

### Sequencing of polymerase chain reaction products to confirm amplification of the regions of interest

Nine of the most prevalent genes (Table 2) were amplified using single PCR and 10 µL of each sample was sent for sequencing at Inqaba Biotech, Pretoria, South Africa. PCR products were purified and concentrated by excising them from a 1% agarose gel using a sterile scalpel and then using a Zymo Research DNA clean and concentrator-5 kit (Epigenetics Company, Irvine, California, USA). Purification was carried out at the National University of Science and Technology (NUST). Sequencing was performed using an automated ABI-Prism 3100 Genetic Analyser (Applied Biosystems, Foster City, USA) according to the manufacturer's instructions. DNA sequence data (chromatographs and sequences) were sent back by email for analysis. Sequence results were analysed using Basic Local Alignment Search Tool (BLAST) in the National Centre for Biotechnology Information (NCBI) databases to confirm the identity of the amplified regions.

## Results

### *Escherichia coli* virulence gene screening

**FIGURE 1:**
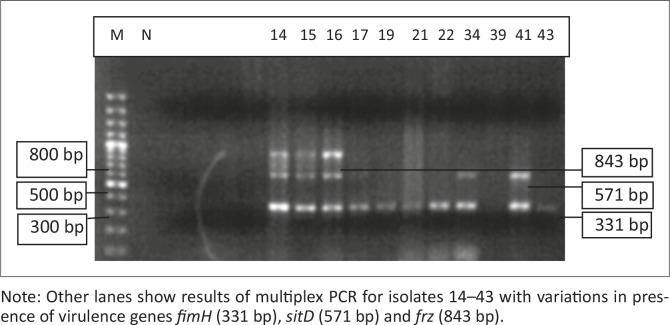
Multiplex 1. Lane M indicates the lane containing the marker, GeneRuler 100 bp Plus DNA Ladder (Thermo Scientific, Waltham, Massachusetts, USA), whilst lane N indicates the negative control. Note: Other lanes show results of multiplex PCR for isolates 14–43 with variations in presence of virulence genes *fimH* (331 bp), *sitD* (571 bp) and *frz* (843 bp).

**FIGURE 2:**
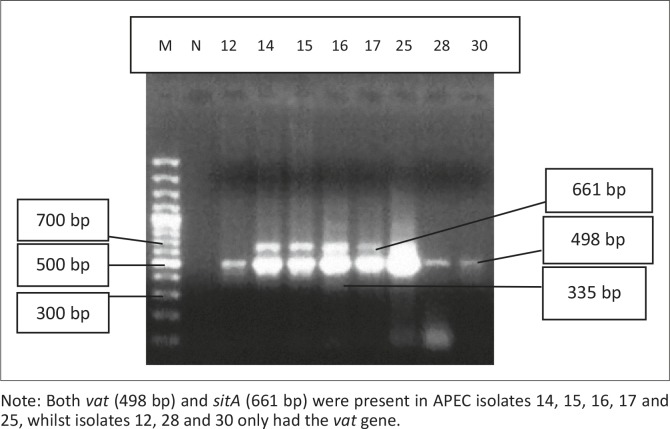
Multiplex 2. Lane M indicates the lane containing the marker, GeneRuler 100 bp Plus DNA Ladder (Thermo Scientific, Waltham, Massachusetts, USA) whilst lane N indicates the negative control. Note: Both *vat* (498 bp) and *sitA* (661 bp) were present in APEC isolates 14, 15, 16, 17 and 25, whilst isolates 12, 28 and 30 only had the *vat* gene.

**FIGURE 3:**
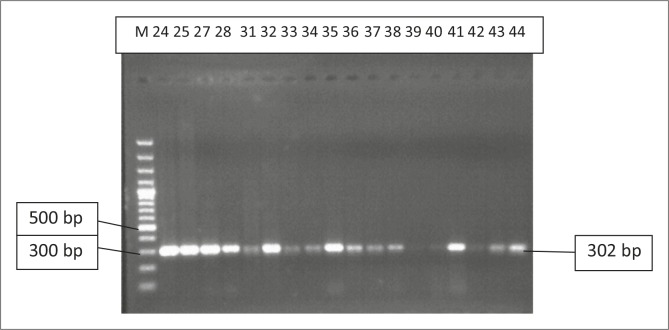
Multiplex 3. Lane M indicates the lane containing the marker, GeneRuler 100 bp Plus DNA Ladder (Thermo Scientific, Waltham, Massachusetts, USA). Isolates 24–44 all had the iutA (302 bp) gene.

**FIGURE 4:**
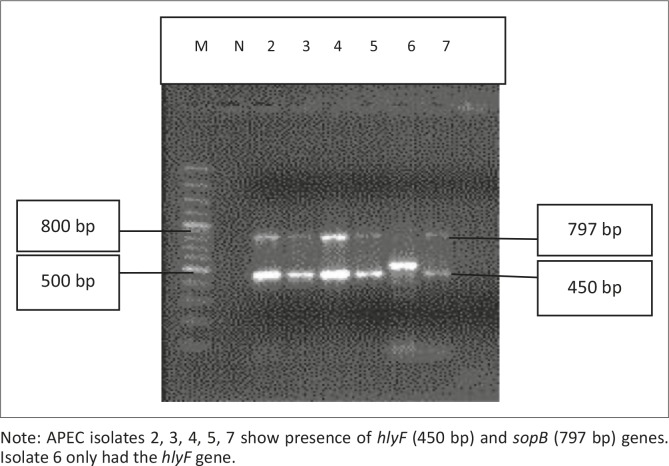
Multiplex 4. Lane M indicates the lane containing the marker, GeneRuler 100 bp Plus DNA Ladder (Thermo Scientific, Waltham, Massachusetts, USA) whilst lane N indicates the negative control. Note: APEC isolates 2, 3, 4, 5, 7 show presence of *hlyF* (450 bp) and *sopB* (797 bp) genes. Isolate 6 only had the *hlyF* gene.

All 45 isolates obtained from the Central Veterinary Laboratories (CVL), Bulawayo were positively identified and confirmed to be *E. coli* through culturing and biochemical tests. After successful DNA isolation and quantification, the DNA of each of the 45 APEC isolates was subjected to four different multiplex PCRs. Each multiplex reaction amplified three APEC virulence gene regions. This was done in order to screen the APEC isolates for 12 virulence associated genes. Figure 1 shows results for multiplex 1, which targeted the *frz, sitD* and *fimH* virulence genes. Some of the APEC isolates (14, 15 and 16) had all three genes present (Figure 1). Multiplex 2 targeted the *kpsM, sitA* and *vat* virulence genes. Only one isolate (isolate 16) had all three genes, whilst isolates 12, 28 and 30 only had the *vat* gene (Figure 2). Figure 3 shows results for multiplex 3, which assayed for the presence of the *ompT, iutA* and *pstB* genes. Most of the APEC isolates had a 302 bp amplicon consistent with the *iutA* gene. Multiplex 4 assayed for the presence of the *sopB, uvrY* and *hlyF* virulence genes. Figure 4 shows the amplification of two bands 450 bp (*hlyF*) and 797 bp (s*opB*) for isolates 2, 3, 4, 5 and 7 and only one band (450 bp) for isolate 6. The most prevalent virulence associated genes in the APEC isolates tested were the *iutA* gene (80%), *fimH* (33.3%), *hlyF* (24.4%) and *sopB* (20%) (Table 2). The data obtained from electrophoresis agarose gels were used to assign virulence gene profiles to each APEC isolate (Table 3).

### Single polymerase chain reactions and sequencing

Nine of the most prevalent genes were successfully amplified using single PCR and produced the predicted sized bands during electrophoresis. To further confirm the identity of the amplicons, the bands obtained through electrophoresis were purified, concentrated and sent for sequencing. The sequences obtained (data not shown) were analysed using BLASTn and BLASTx and the amplicons were confirmed to be part of the genes of interest (data not shown).

## Discussion

In this study, 45 *E. coli* isolates were obtained from chickens with confirmed cases of colibacillosis and screened for 12 virulence genes commonly associated with pathogenicity in APEC. Some of the virulence factors investigated have only been discovered recently. Ninety-three percent of the *E. coli* isolates in this study had at least one virulence gene, suggesting that the isolates used could have been APEC.

The *iutA* gene had the highest prevalence at 80% (Table 2). This gene has been well characterised and is one of the five genes of the aerobactin operon. It encodes an outer membrane protein involved in the high-affinity binding of Fe^3-^aerobactin and can be plasmid located (Johnson *et al*. 2006) or chromosomally encoded in some APEC strains (Schouler *et al*. 2012). The aerobactin system plays a role in the persistence and generation of lesions in APEC-infected chickens. The present findings are in agreement with other studies that found an equally high prevalence of the *iutA* gene in APEC isolates. These include the studies of Rodriguez-Siek *et al.* (2005), Johnson *et al.* (2008), and Schouler *et al.* (2012), who found prevalence rates of 80.2%, 80.8% and 82.7% respectively. The Type 1 fimbrial adhesion gene (*fimH*) had a prevalence of 33.3% in this study. *FimH* is thought to contribute to the protection of *E. coli* from host heterophils (Mellata *et al*. 2003). Its role in the virulence of APEC strains remains controversial, with conflicting reports (Li *et al*. 2005). Other studies have found higher occurrences of the *fimH* gene, notably that of Rodriguez-Siek *et al*. (2005), who found a prevalence of 98.1% for this gene in 524 APEC isolates. Interestingly, in a study by Van der Westhuizen and Bragg (2012) using 10 Zimbabwean APEC isolates, all 10 had the *fimH* gene present. The *hlyF* gene has been shown to contribute to iron uptake (Morales *et al*. 2004; Williams & Warner 1980). *HlyF* has been well documented in chickens suffering from colibacillosis (Van der Westhuizen & Bragg 2012). The *hlyF* gene had a prevalence rate of 24.4% in the current study (Table 2). This disagrees with several studies that have found higher prevalence rates (Johnson *et al*. 2008; Kafshdouzan *et al*. 2013). Another study, however, found a lower prevalence rate of a related gene, *hlyE* (Jin *et al*. 2008). The plasmid partitioning protein encoded by *sopB* is common in various plasmids associated with virulence characteristics in APEC (Van der Westhuizen & Bragg 2012). The *sopB* gene had a prevalence rate of 20%, which agrees with results from the study carried out on 10 Zimbabwean APEC isolates by Van der Westhuizen and Bragg (2012), as 30% of their isolates had the *sopB* gene. The vacuolating autotransporter toxin (*vat*) gene, which has been shown to induce cytotoxic effects in host cells (Parreira & Gyles 2003), was present in 17.8% of the isolates. This low prevalence rate agrees with other studies, in which detection rates of 39.8% (Ewers *et al*. 2007), 25.5% (Jin *et al*. 2008) and 33.4% (Johnson *et al*. 2008) were found in APEC. The *sitA* and *sitD* genes are part of the *sitABCD* system. Gene *sitA* encodes a periplasmic binding protein of the *sitABCD* transport system, which is involved in iron and manganese transport and can be both chromosomally and plasmid located (Mellata, Touchman & Curtiss 2009; Sabri, Léveillé & Dozois 2006; Schouler *et al*. 2004). *SitABCD* has been shown to play a role in virulence (Sabri *et al*. 2008). The prevalence of the *sitA* (11.1%) and *sitD* (13.3%) genes (Table 2) is comparable to that found by Van der Westhuizen and Bragg (2012) but differs from those reported by Schouler *et al*. (2012) and Johnson *et al*. (2008) for the *sitA* gene and by Ewers *et al*. (2007) for the *sitB* gene. The presence of these two genes agrees with the results of other scholars who believe that in *E. coli* *sitABCD*-encoding genes are associated with clinical strains isolated from extra-intestinal infections from poultry and human urinary tract infections (Rodriguez-Siek *et al.* 2005; Schouler *et al.* 2004). The *frz* operon was present in 8.9% of the *E.coli* isolates (Table 2). Work by Rouquet *et al*. (2009) suggested that the gene products from the *frz* operon are used by *E.coli* to promote growth in serum during oxygen-restricted conditions. A link between the expression of this locus and *E. coli* pathogenic abilities was confirmed by experiments showing its role in promoting bacterial fitness under stressful conditions, including oxygen restriction or the late stationary phase of growth, and in promoting growth in chicken serum or the intestinal tract during *in vivo* competition assays (Schouler *et al*. 2012). The results of this study agree with those of Van der Westhuizen and Bragg (2012) with regard to the Zimbabwean isolates they worked on; however, they differ from work carried out by Schouler *et al*. (2012) in which they found a prevalence of 53.4% for the *frz* operon in 352 APEC isolates.

A transcriptional regulator of iron uptake genes in APEC, *uvrY* (Li *et al*. 2005) has only recently been used to screen for APEC using multiplex PCR (Van der Westhuizen & Bragg 2012). In this study, this gene had a low prevalence rate of 4.4% (Table 2). This differs from the 100% reported by Van der Westhuizen and Bragg (2012) for the 10 Zimbabwean isolates they studied. The *ompT*, *pstB* and *kpsM* genes all had a low prevalence rate of 2.2%. The *ompT* gene encodes the episomal outer membrane protease that cleaves colicins (Cavard & Lazdunski 1990). Other studies on APEC have shown higher detection rates of this gene (Johnson *et al.* 2008; Kafshdouzan *et al*. 2013; Rodriguez-Siek *et al.* 2005). The *kpsM* gene encodes proteins required for translocation of *E. coli* group II capsular polysaccharide across the inner membrane (Clarke, Pearce & Roberts 1999). Prevalence rates of the *kpsM* gene reported for APEC isolates have tended to be low: 15.7% (Johnson *et al.* 2008), 15.8% (Rodriguez-Siek *et al*. 2005) and 0% for Zimbabwean isolates (Van der Westhuizen & Bragg 2012). The *pstB* gene, which is part of the *pst*SCAB operon, has been shown to increase resistance to polymyxin, rabbit serum and acid shock (Lamarche *et al*. 2005). The *pstB* gene contributes to virulence but is still relatively new in the diagnostic context (Lamarche *et al*. 2005). As a result very little has been published on this virulence gene in APEC.

Virulence profiles were generated for each *E. coli* isolate used in this study (Table 3). The APEC isolates were profiled as 62.2% fitting profile A, 31.1% profile B and 6.7% profile C. None of the isolates had more than seven virulence genes. These findings agree with those of Van der Westhuizen and Bragg (2012). They found the Zimbabwean APEC isolates to have fewer virulence genes (with most having less than 11/18 virulence genes studied) than the South African APEC isolates, which all had between 12 and 18 virulence genes. The low number of virulence genes in the isolates assayed in this study could be a result of the fact that these isolates possess other virulence genes that were not screened for. Numerous studies have demonstrated that virulence genes are rarely all present in the same isolate. Different isolates may harbour different associations of virulence genes and belong to specific subpathotypes, with each subpathotype characterised by the type of lesions it produces in poultry with avian colibacillosis (Maturana *et al*. 2011; Olsen *et al*. 2012). Another possibility is that the isolates used in this study belong to strains that are not highly pathogenic and that environmental or other disease factors caused stress in the hosts, which allowed them to be infected and led to colibacillosis (Van der Westhuizen & Bragg 2012). The risk for colibacillosis is known to increase with increasing infection pressure in the environment. Good housing, hygiene and avoiding overcrowding are very important in reducing infection rates (Saidi *et al*. 2013). Another reason, although less likely, could be that the strains used in this study were not the causative agents in the confirmed colibacillosis cases. This is because isolation of an *E. coli* strain from a pathological lesion is not a sufficient criterion to classify it as a pathogen (Schouler *et al*. 2012).

## Limitations

Potential biases in this study are related to the relatively small sample size of *E. coli* isolates used. In addition, *in vivo* tests of the isolates to determine their pathogenicity could have improved the study. In retrospect, more virulence genes could have been screened and positive controls for all 12 genes studied could have been used.

### Recommendations

Screening for more virulence factors using a larger sample size could provide more definite conclusions. Also, a pathogenicity test of isolates using the 1-day-old chick lethality test to correlate virulence profiles with pathogenicity is recommended. It might be interesting to study APEC from other poultry sources like ducks, geese and turkeys to determine relationships with APEC-causing colibacillosis in chickens.

## Conclusion

The study revealed that the virulence profiles of Zimbabwean APEC isolates may be different from those reported in other studies, which generally show a high prevalence of the virulence genes we investigated in APEC isolates causing colibacillosis in poultry (Ewers *et al*. 2007; Johnson *et al*. 2008; Kafshdouzan *et al*. 2013). This suggests that other virulence genes not investigated in the present study may be important in virulence of Zimbabwean APEC isolates or, more likely, that these isolates are less pathogenic and rely on environmental factors and stress in hosts to establish infection.

## Appendix 1

**TABLE A1:** Primers used for amplifying regions in avian pathogenic *Escherichia coli* virulence genes.

Primer	Primer name	Primer sequence (5’ – 3’)	Name of targeted region/gene	Source of primer sequence	Expected amplicon size (bp)
Forward primer	*sitA*-F	CGCAGGGGGCACAACTGAT	SitABCD system	Sabri *et al*. (2008)	661
Reverse primer	*sitA*-R	CCCTGTACCAGCGTACTGG			
Forward primer	*sitD*-F	CTGTGCGCTGCTGTCGGTC	SitABCD system	Sabri *et al*. (2008)	571
Reverse primer	*sitD*-R	GCGTTGTGTCAGGAGTAC			
Forward primer	*ompT*-FP	TCATCCCGGAAGCCTCCCTCACTACTAT	Episomal outer membrane protease	Johnson *et al*. (2008)	496
Reverse primer	*ompT*-RP	TAGCGTTTGCTGCACTGGCTTCTGATAC			
Forward primer	*fimH*-FP	GGATAAGCCGTGGCCGGTGG	Type 1 fimbrial adhesin fimH	Van der Westhuizen and Bragg (2012)	331
Reverse primer	*fimH*-RP	CTGCGGTTGTGCCGGAGAGG			
Forward primer	*frz*-FP	GAGTCCTGGCTTGCGCCGTT	frz operon	Van der Westhuizen and Bragg (2012)	843
Reverse primer	*frz*-RP	CCGCTCCATCGCAGCCTGAA			
Forward primer	*hlyF*-FP	GGCCACAGTCGTTTAGGGTGCTTACC	Putative avian haemolysin	Johnson *et al*. (2008)	450
Reverse primer	*hlyF*-RP	GGCGGTTTAGGCATTCCGATACTCAG			
Forward primer	*iutA*-FP	GGCTGGACATCATGGGAACTGG	Aerobactinsiderophore receptor	Johnson *et al*. (2008)	302
Reverse primer	*iutA*-RP	CGTCGGGAACGGGTAGAATCG			
Forward primer	*kpsM*-FP	CAGCCTCGCGGCTTAGCTCC	Capsule-protein transport of polysaccharides	Van der Westhuizen and Bragg (2012)	335
Reverse primer	*kpsM*-RP	TGCACGCGCACTGCTTGAGA			
Forward primer	*vat*-FP	CGCTTCAGGTGCGCTGACCA	Vacuolating autotransporter toxin	Van der Westhuizen and Bragg (2012)	498
Reverse primer	*vat*-RP	AAGGGAGACGATGCCCGCCT			
Forward primer	*pstB*-FP	CGCGCTCGTCCATGTCAGCA	PstSCAB system	Van der Westhuizen and Bragg (2012)	198
Reverse primer	*pstB*-RP	CGGAACAGCGTGCGGAAGGT			
Forward primer	*uvrY*-FP	TGAGTGCGATTCGTTCTGTC	APEC virulence regulator	Herren *et al*. (2006)	286
Reverse primer	*uvrY*-RP	TCTCCGCATTACACAGACCA			

Note: Please see the full reference list of the article Mbanga, J. & Nyararai, Y.O., 2015, ‘Virulence gene profiles of avian pathogenic *Escherichia coli* isolated from chickens with colibacillosis in Bulawayo, Zimbabwe’, *Onderstepoort Journal of Veterinary Research* 82(1), Art. #850, 8 pages. http://dx.doi.org/10.4102/ojvr.v82i1.850, for more information.
